# Selenite as a dual apoptotic and ferroptotic agent synergizes with EGFR and KRAS inhibitors with epigenetic interference

**DOI:** 10.1186/s13148-023-01454-4

**Published:** 2023-03-02

**Authors:** Lok Seng Chan, Johnson Liu, Molly S. C. Li, Lili Li, Qian Tao, Tony S. K. Mok

**Affiliations:** Cancer Epigenetics Laboratory, Department of Clinical Oncology, State Key Laboratory of Translational Oncology, Sir YK Pao Center for Cancer and Li Ka Shing Institute of Health Sciences, The Chinese University of Hong Kong, Prince of Wales Hospital, Room 315, Sir Yue-Kong Pao Center for Cancer, Shatin, Hong Kong

**Keywords:** Selenite, Lung adenocarcinoma, Unfolded protein response, Ferroptosis, Osimertinib

## Abstract

**Background:**

Selenium, an essential trace element, has previously been investigated as a pro-apoptotic and DNA demethylation agent. It sensitizes the response to chemotherapy in patients who were refractory to cytotoxic agents. Meanwhile, ferroptosis is a novel approach to cancer treatment by triggering cell death and reversing drug resistance. The role of selenium in treating cancer cells harboring druggable oncogenic alterations and its underlying mechanism are largely unknown.

**Results:**

We treated lung adenocarcinoma cell lines—EGFR-mutant H1975 (H1975 ^EGFR p.L858R and p.T790M^) and KRAS-mutant H358 (H358 ^KRAS p.G12C^), with sodium selenite to examine its effect on cell apoptosis, ferroptosis, and DNA methylation, as well as its interaction with existing targeted therapy, osimertinib, and adagrasib. We observed selenite to be a dual apoptotic and ferroptotic agent on lung cancer cells, associated with the activation of p38-ATF4-DDIT3 axis in the unfolded protein response. Ferroptosis induction was more remarkable in H1975 than H358. Selenite also altered cellular DNA methylation machinery through downregulating DNMT1 and upregulating TET1, though not as a major mechanism of its activity. Low-dose selenite synergized with osimertinib in EGFR-mutant H1975, and with adagrasib in KRAS-mutant H358, with stronger synergism observed in H1975.

**Conclusion:**

These results suggest that selenite is a potential apoptotic and ferroptotic drug candidate for the treatment of especially EGFR- and potentially KRAS-mutant lung cancer.

**Supplementary Information:**

The online version contains supplementary material available at 10.1186/s13148-023-01454-4.

## Background

Lung cancer is a leading cause of cancer death worldwide. Targeted therapy is well established as the first- or second-line treatment for patients with metastatic lung adenocarcinoma (LUAD) harboring actionable oncogenic alterations. Sensitizing EGFR mutations, such as exon 19 deletion and exon 21L858R mutation, are found in 15% of Caucasians and up to 50% of Asians with LUAD [1, 2]. Meanwhile, KRAS G12C mutation, the recently and only approved druggable KRAS target, accounts for 15% of Caucasians with LUAD [3]. The presence of such mutations predicts treatment benefit with tyrosine kinase inhibitors (TKI) like osimertinib in EGFR mutations [4], and KRAS inhibitors such as adagrasib in KRAS mutation [5].

Selenium is an essential trace element for the synthesis of selenoproteins, including five glutathione peroxidases (GSH) and three thioredoxin reductases, which protect cells from oxidative stress and cell death [6]. Moreover, at pharmacological doses, selenium compounds, such as sodium selenite, were demonstrated to possess multiple antineoplastic properties. In vitro, selenium compounds induced reactive oxidative species (ROS)-dependent endoplasmic reticulum (ER) stress-mediated cell death, including normal endothelial, liver and testis cells, and colorectal, prostate and oral squamous cell carcinoma [7–13].

As a novel form of programmed cell death, ferroptosis is distinct from apoptosis, necroptosis, and autophagy. The hallmark of ferroptosis is the accumulation of lipid peroxides. The process is often initiated by impaired removal of ROS by defense systems like the glutathione/GSH system, increased ROS generation, or altered iron metabolism [14]. In particular, GSH utilizes glutathione as a substrate to remove ROS, while glutathione synthesis involves the conjugation of cysteine and glutamate and then the addition of glycine [15]. Therefore, cytoplasmic levels of these amino acids are essential for cellular redox balance. Currently, ferroptosis inducers are classified by their mechanism of action, including the inhibition of SLC7A11, which constitutes the cysteine/glutamate antiporter, to cause depletion of intracellular cysteine and glutathione, direct inhibition of GPX4 catalytic activity, depletion of GPX4 and ubiquinone, as well as by the oxidation of iron and subsequent indirect GPX4 inactivation [16]. Meanwhile, there is an intricate relationship between ER stress and ferroptosis. Redox imbalance can lead to misfolding of proteins and elicit an unfolded protein response (UPR) [17]. Upon the inhibition of cystine/glutamate antiporter, upregulation of CHAC1 has been observed [18]. Other studies showed that CHAC1 degrades GSH to further enhance the ferroptotic effect [19]. Meanwhile, ATF4, a transcription factor that binds the cAMP response elements, is expressed in different cellular stresses to regulate UPR [20, 21]. Lastly, DDIT3/CHOP is a pro-apoptotic transcription factor controlled by ATF4 that promotes the expression of BH3-only proteins including PUMA, NOXA, and BIM, to promote apoptosis as well as *CHAC1* to reinforce the ferroptotic process [19, 22, 23].

Selenite also induces cell cycle arrest and apoptosis in multiple tumors [24, 25]. Additionally, selenite downregulates DNMT1, causing re-expression of certain tumor suppressor genes (TSGs) by promoter demethylation [26–28], such as restoration of *GSTP1*, *APC*, and *CSR1* in prostate cancer, and *VHL* in colon cancer [29, 30]. However, there has been little focus on both lung cancer and tumors harboring driver oncogene mutations. Therefore, the anti-tumor effect of selenium in oncogenic mutant LUAD cells and its underlying mechanisms remain unclear.

Clinically*, *in vivo data have shown that low selenium status is associated with multiple cancers [31]. Indeed, selenium has failed to prevent secondary primary tumors in patients with resected stage I non-small cell lung cancer and demonstrated an insignificant chemopreventive effect on lung cancer [32–35]. However, in the phase 1 SECAR trial, the addition of selenium to chemotherapy was tolerable and resensitized the tumor to chemotherapy in patients who developed chemotherapy resistance [36], suggesting a potential role of selenium in cancer treatment instead of chemoprevention. However, the synergism between selenite and targeted therapy has not been well understood when compared with that of chemotherapy.

In this study, we sought to elucidate the antineoplastic properties of selenium on EGFR- and KRAS-mutant LUAD cells, by exploring its effect on apoptosis, DNA methylation machinery, transcriptomic landscape, and ferroptosis. We also explored its potential to synergize EGFR TKI and KRAS inhibitors.

## Results

### Selenite inhibited growth and induced apoptosis in LUAD cells

**Fig. 1:**
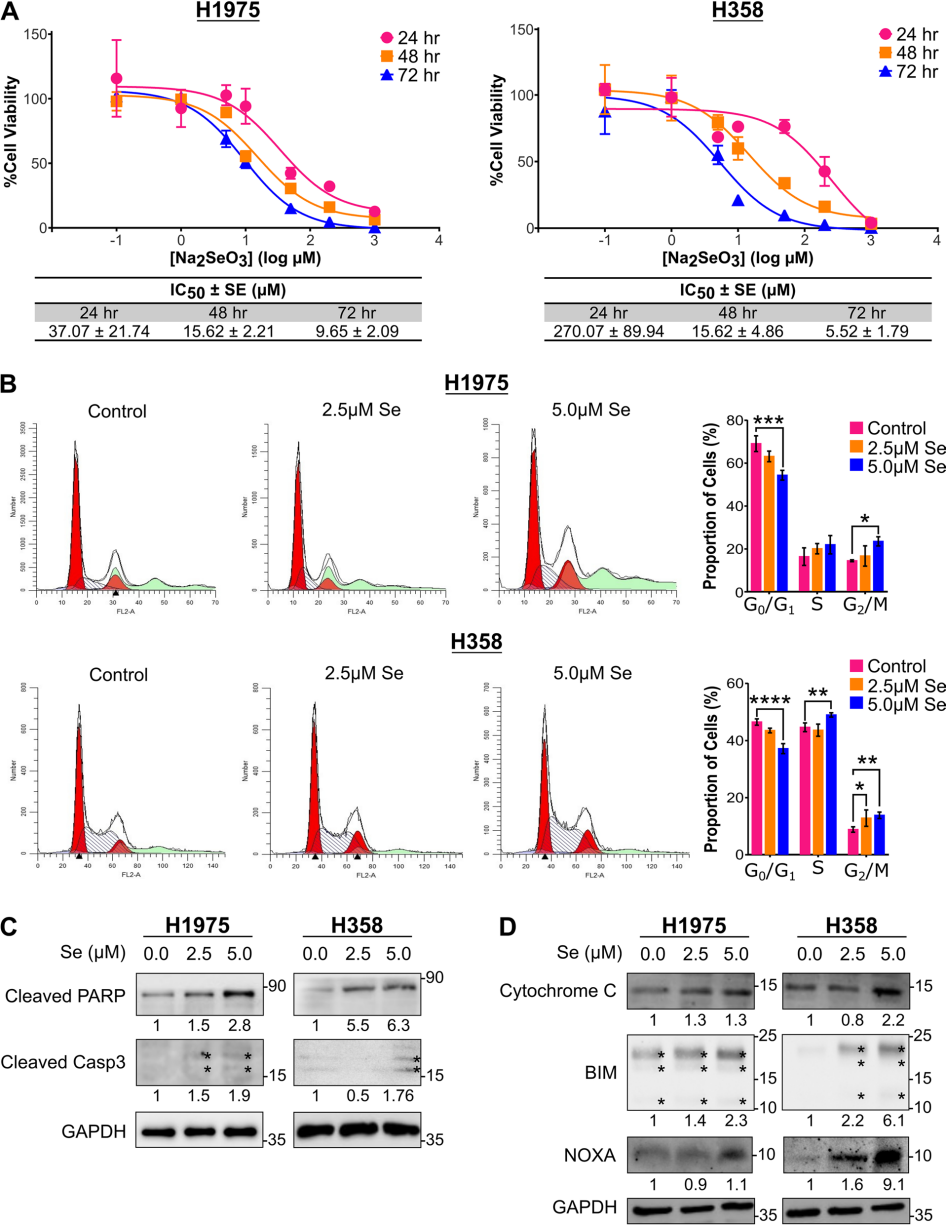
Selenite-induced cell cycle arrest and cell death. **A** Cell viability and IC_50_ of H1975 and H358 treated with selenite at 24, 48, and 72 h were assessed by MTS assay. **B** Flow cytometry revealed cell cycle arrest induced by sodium selenite at 72 h. **C** Western blot analysis of cleaved PARP and cleaved caspase 3 in 72-h selenite-treated cells demonstrated apoptosis. **D** Western blot analysis showed upregulation of cytochrome C, BIM, and NOXA, representative markers of the intrinsic pathway of apoptosis caused by 72-h selenite treatment

To evaluate the anti-tumor effect of selenite on oncogene mutant LUAD cell lines, H1975 harboring EGFR^L858R/T790M^ mutation and H358 harboring KRAS^G12C^ mutation were used. We first examined cell viability under selenite treatment at 24, 48, and 72 h by MTS assay. Over 90% anti-viability was achieved in both cells at all time points. The IC_50_ values in selenite at 48 h were H1975: 15.62 ± 2.21, H358: 15.62 ± 4.86 and at 72 h H1975: 9.65 ± 2.09 and H358: 5.52 ± 1.79 (Fig. 1A).

We next determined the effect of selenite on cell cycle using propidium iodide staining. The proportion of cells in G2/M phase increased from 14.5 to 16.7% (2.5 µM) to 23.5% (5.0 µM) and from 8.9 to 12.8% (2.5 µM) to 13.8% (5.0 µM), respectively, in H1975 and H358, accompanied by a decrease in cells in the G0/G1 phase (Fig. 1B). These data are indicative of a G2/M arrest although further molecular evidence is warranted.

We further investigated whether selenite could induce apoptosis in EGFR- and KRAS-mutant LUAD cells. 72-h selenite treatment produced a dose-dependent increase in cleaved PARP in both cells, as well as a dose-dependent but mild increase in cleaved caspase 3 in H1975 but not H358 (Fig. 1C). We further tested the expression of components of the intrinsic apoptosis pathway. Upregulation of cytochrome C, BIM, and NOXA in both cells confirmed that the pro-apoptotic function of selenite was via the intrinsic mitochondrial pathway (Fig. 1D). Selenite treatment did not alter the cleavage of caspase 8, suggesting that the extrinsic pathway was no involved (Additional file 1: Fig. S1A). These data suggest that selenium triggered apoptosis via the intrinsic pathway.

### Effect of sodium selenite on DNA methylation machinery and transcriptome

**Fig. 2:**
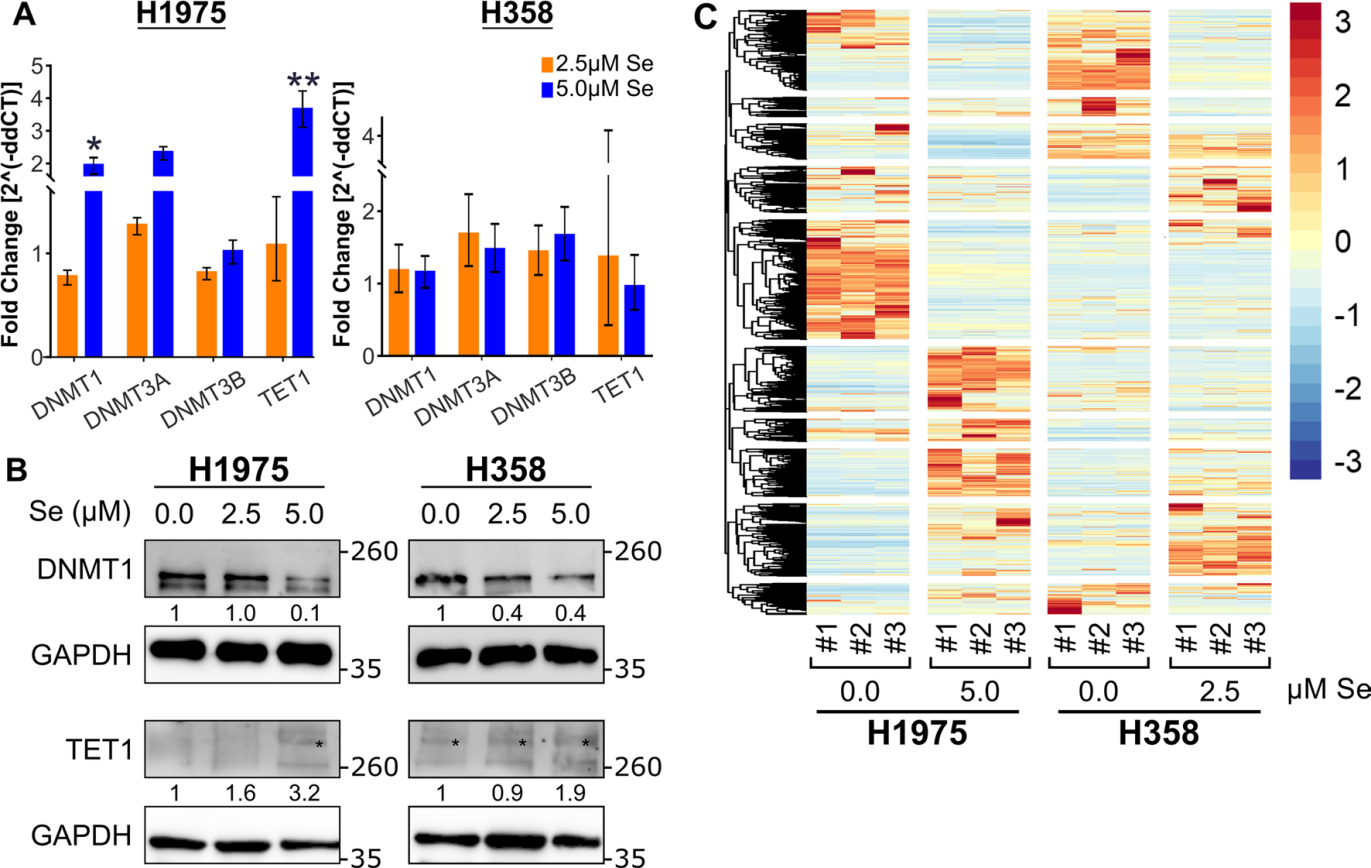
Selenite treatment altered DNA methylation machinery mainly at the protein level and altered the transcriptomic landscape. **A** The mRNA levels of DNA methylation machinery treated for 72 h were assessed by RT-qPCR and demonstrated upregulation in DNMT1 and TET1 in H1975 only. **B** Western blot analysis of H1975 and H358 cell lysate treated for 72 h demonstrated downregulation in DNMT1 and upregulation in TET1. **C** Heatmap of the differentially expressed genes

Selenite has been suggested to be a potential demethylation agent in some tumor cells. We next examined its effects on the mRNA expression and protein levels of DNA methylases and demethylase in EGFR- and KRAS-mutant LUAD cells. At the mRNA level, 5.0 µM selenite caused upregulation in the mRNA expression of DNMT1, DNMT3a, and TET1 in H1975, but both 2.5 µM and 5.0 µM selenite failed to cause any significant change in the expression of DNMT1, DNMT3A, DNMT3B, and TET1 in H358 (Fig. 2A). Protein expression of DNMT1 after selenite treatment was further studied, and downregulation of DNMT1 occurred at 2.5 µM in H358 and 5.0 µM in H1975 (Fig. 2B). TET1 protein expression was detectable only at 5.0 µM in H1975 and was upregulated at 2.5 µM and 5.0 µM in H358.

As epigenetic agents can reprogram cell transcriptome, RNA sequencing (RNA-seq) was performed to determine the effect of selenite on genome-wide cellular gene expression changes. H1975 and H358 were treated at 5.0 µM and 2.5 µM selenite, respectively, at which DNMT1 was downregulated. Significance was determined by an absolute value of log_2_ fold change greater than 0.5 and a q-value of less than or equal to 0.05. At transcript resolution, there were 4,548 and 1745 differentially expressed genes (DEGs) identified in H1975 and H358, respectively (Additional file 1: Fig. S1B). Selenite altered the transcriptomic landscape of both cell lines (Fig. 2C). Gene enrichment in cell cycle checkpoints and ATR signaling using the Reactome database echoed our previous findings of cell cycle arrest (Additional file 1: Fig. S1C).

### Selenite altered mRNA expression of genes involved in MAPK Signaling

**Fig. 3:**
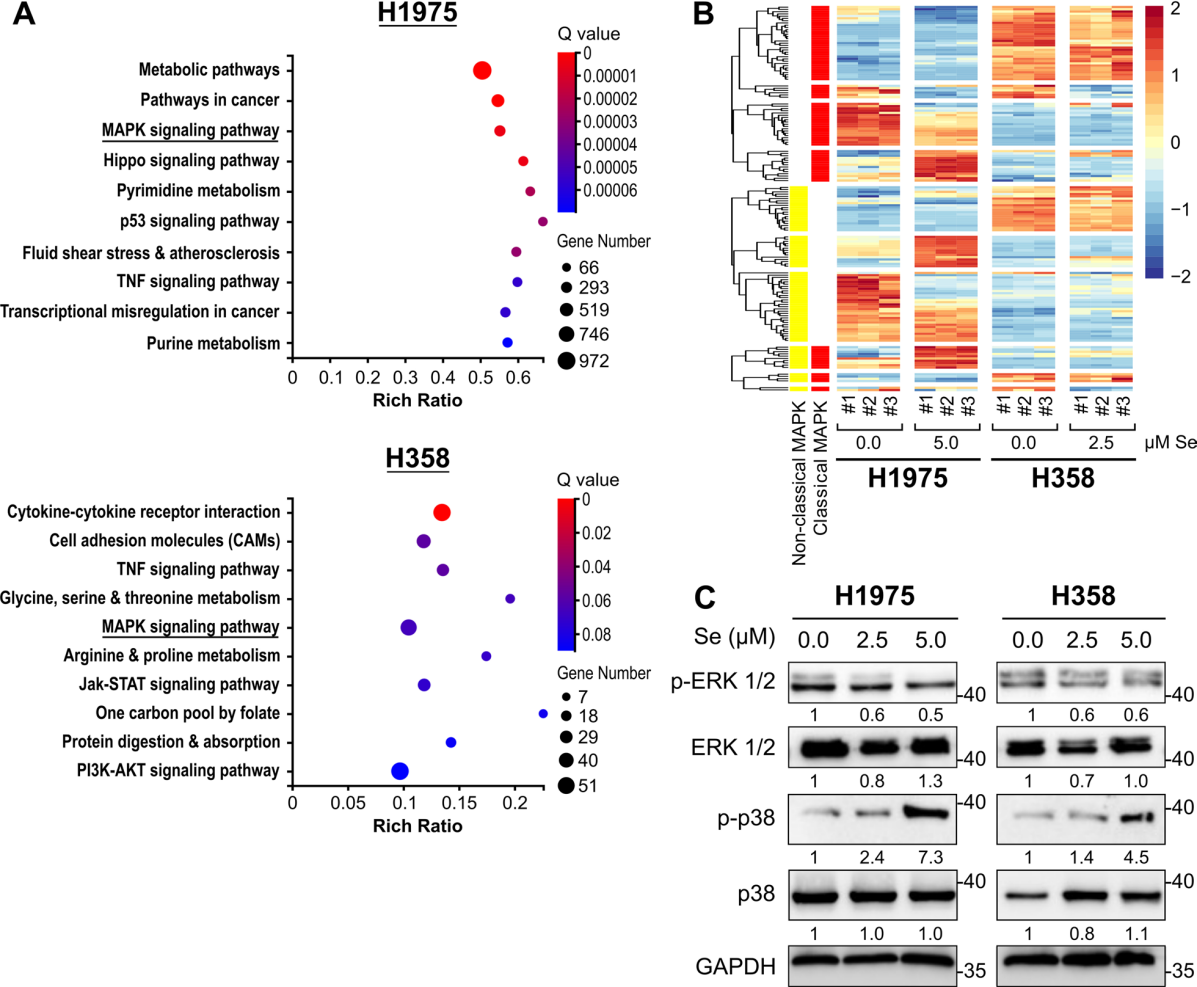
Selenite treatment was associated with dysregulated classical and non-classical MAPK signaling. **A** KEGG enrichment of the differentially expressed genes at the transcript resolution of H1975 treated with or without 5.0 µM selenite and H358 treated with or without 2.5 µM selenite for 72 h. **B** Heatmap of the sequencing data revealed dysregulated classical and non-classical MAPK pathways mainly in H1975 treated with 72 h 5.0 µM selenite. **C** Western blot analysis of H975 and H358 on ERK and p38 signaling treated for 72 h

To further analyze the transcriptomic landscape changes of the two cell lines, we performed KEGG pathway enrichment analysis on the DEGs and representative pathways were identified (Fig. 3A). MAPK signaling and several metabolic pathways were dysregulated in both cells. Further analysis was performed on MAPK signaling because it constitutes the downstream EGFR and RAS signaling pathway and drives oncogenesis. Annotated heatmap illustrated that MAPK-related genes were more dysregulated in H1975 than H358 after selenite treatment. The dysregulation took place in both classical MAPK, or ERK, signaling, and non-classical MAPK signaling, which includes the p38 MAPK signaling (Fig. 3B). Western blot revealed only minor downregulation in phospho-ERK1 levels in H1975 (Fig. 3C). For p38 MAPK, an increase in phosphorylation level was observed in both cells, suggesting the presence of cell stress (Fig. 3C). Therefore, the cytotoxicity of selenite could be attributed more to cell stress and only less to its inhibitory effect on cell survival pathways like ERK1/2.

### Selenite is associated with oxidative stress in H1975 and DNA damage in H358

**Fig. 4:**
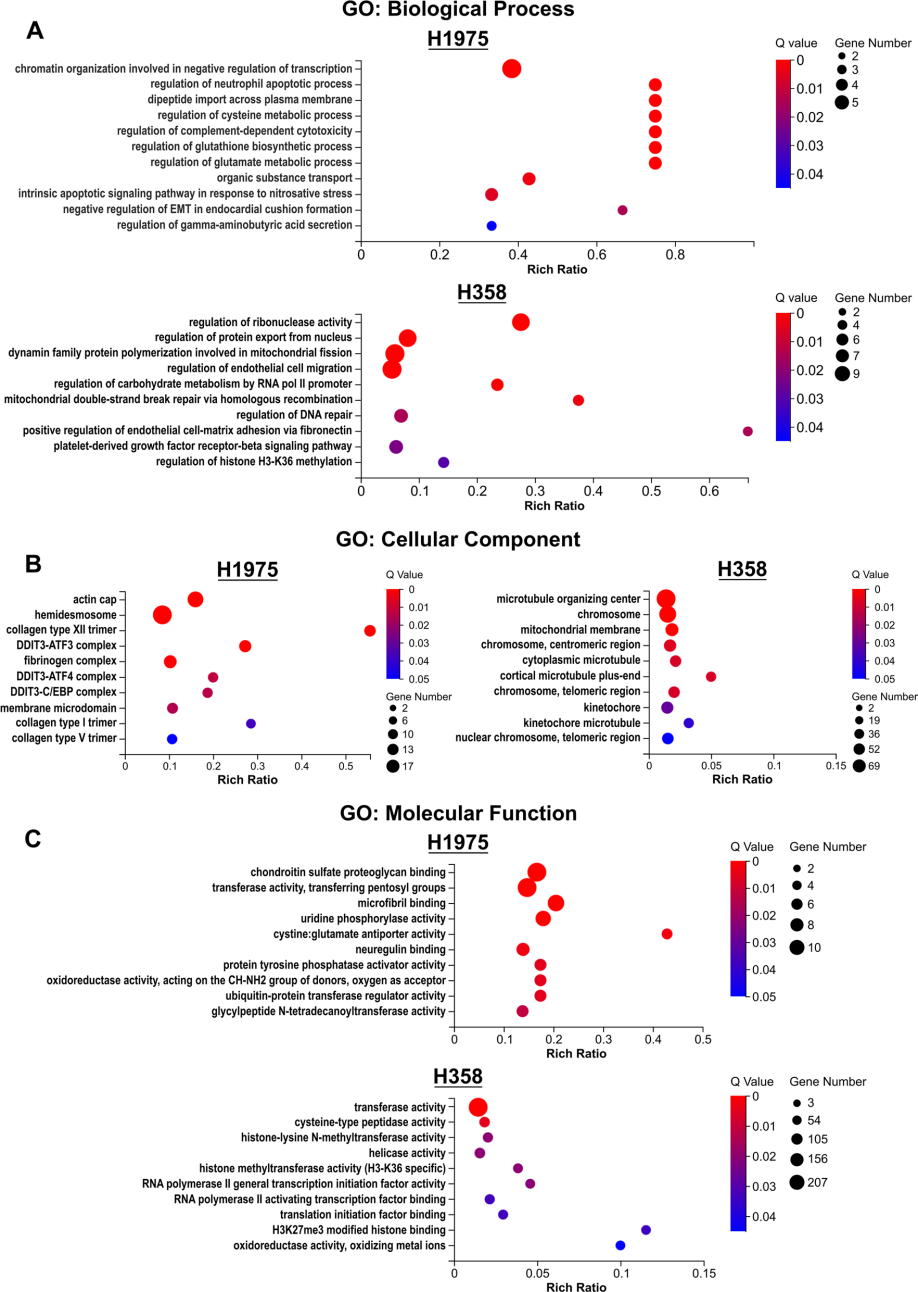
Selenite treatment was associated with enriched gene ontology (GO) terms related to ER stress and glutathione synthesis in H1975. GO enrichment analysis of the term **A** GO: biological process, **B** cellular components, and **C** molecular function of H1975 treated with or without 5.0 µM selenite and H358 treated with or without 2.5 µM selenite for 72 h

To better characterize the type of cellular stress, enrichment analyses based on the gene ontology (GO) terms were performed for DEGs with a higher absolute log2 fold change of greater than or equal to 1.5. Enrichment of GO biological processes showed that in EGFR-mutant H1975, apoptosis and many metabolic processes, including glutathione synthesis, glutamate, and cysteine metabolic processes, were affected (Fig. 4A). In particular, dysregulated metabolic processes in H1975 were highly related to glutathione synthesis. Not only is glutathione synthesized from cysteine and glutamate [15], the enrichment of intrinsic apoptotic signaling pathway in response to nitrosative stress reflected the disturbed redox balance and subsequent apoptosis (Fig. 4A). This was consistent with previous data where the intrinsic apoptotic pathway was activated in selenite-treated cells (Fig. 1D). In H358, these findings were not observed. Instead, processes involved in DNA damage and repair were more pronounced (Fig. 4A). For enrichment using GO cellular component terms, DDIT3 complexes and microtubule-related components were highly enriched in H1975 and H358, respectively (Fig. 4B). Enrichment of GO molecular function demonstrated dysregulation of the cysteine/glutamate antiporter in H1975, which echoed the enriched biological processes involving glutathione synthesis (Fig. 4C). Oxidoreductase activities were also enriched in both cell lines (Fig. 4C). In summary, selenite appeared to act as a traditional chemotherapeutic agent such as by causing DNA damage or affecting the microtubules in H358, whereas it was highly associated with redox balance and ER stress in H1975.

Lastly, gene enrichment of both KEGG and GO terms revealed no significant enrichment in pathways or biological process involving DNA methylation, cellular component involving maintenance of DNA methylation, or molecular function of DNA methylase (Fig. 3A, 4A–C). Interestingly, H3K36 and H3K27 methylation appeared dysregulated in H358 (Fig. 4C). Thus, the limited data hinted a less significant role of epigenetics than cellular stress in selenite-treated oncogenic mutant LUAD.

### Selenite-induced unfolded protein response and ferroptosis

**Fig. 5:**
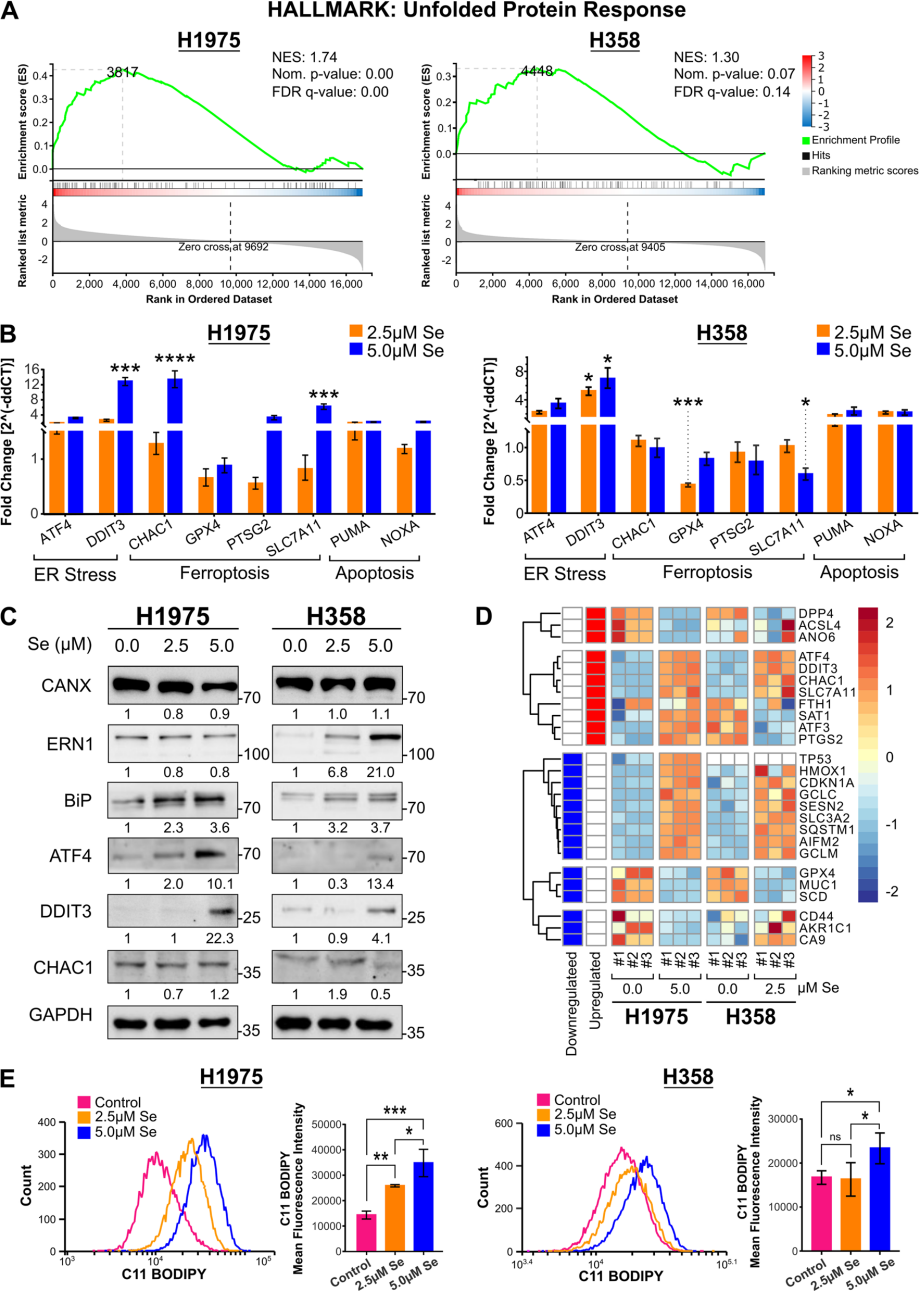
Selenite treatment caused unfolded protein response (UPR) and lipid peroxidation-associated ferroptosis. **A** GSEA analysis of the RNA-seq data at the gene resolution revealed significant UPR in H1975 treated with 5 µM selenite but not in H358 treated with 2.5 µM selenite. **B** RT-qPCR results of both cell lines treated for 72 h at 2.5 µM or 5.0 µM selenite confirmed the findings in transcriptome sequencing related to enriched UPR and ferroptosis pathway. **C** Western blot analysis of representative protein markers of UPR confirmed its activation after selenite treatment of 72 h. **D** Heatmap of the DEGs that was documented to show expression changes in the validated database of FerrDB demonstrated alteration in ferroptosis-related gene mRNA expression 38. **E** C-11 BODIPY staining in cells after 72-h selenite treatment showed an increased level of lipid peroxidation in H1975 and H358

Given that the ER stress was likely involved in H1975, we wanted to better characterize its nature by performing a gene set enrichment analysis on the RNA-seq data at gene resolution. The analysis revealed significant upregulation in genes related to unfolded protein response (UPR) in H1975, but such upregulation was not statistically significant in H358 (Fig. 5A). This might be due to the difference in selenite concentration, so we verified the expression of representative genes which are highly associated with both ER stress and ferroptosis using 2.5 µM and 5.0 µM selenite in both two cell lines.

We detected mRNA expression after selenite treatment by RT-qPCR. A significant upregulation of the ATF4-DDIT3 axis and their downstream genes, namely *CHAC1* and *NOXA*, as well as the anticipated compensatory upregulation of SLC7A11 in ferroptosis [37], were observed in H1975 (Fig. 5B). However, such response was not statistically significant in H358; instead, *GPX4* downregulation and *SLC7A11* downregulation were observed at 2.5 µM and 5.0 µM, respectively (Fig. 5B). Western blot further confirmed the activation of UPR. In H1975, selenite treatment upregulated BiP, ATF4, and DDIT3 in a dose-dependent manner. In H358, apart from these mediators, selenite treatment also upregulated ERN1. However, CHAC1 expression remained unchanged in H1975 and downregulated in H358 when treated with 5 µM selenite (Fig. 5C). This suggested that any ferroptotic response would be independent of CHAC1-mediated degradation of GSH.

Showing a strong association with ferroptosis, the RNA-seq data were plotted to the validated genes in the FerrDb, a database of ferroptosis regulators and markers [38]. The annotation of the heatmap denoted the expected gene expression changes during ferroptosis [38]. Both cells showed a similar pattern of dysregulated transcriptome related to ferroptosis (Fig. 5D). Lastly, we examined the level of lipid peroxidation as a measurement of ferroptosis. 72-h selenite treatment produced a dose-dependent increase in lipid peroxidation levels in both H1975 and H358. Lipid peroxidation was nearly doubled in 5.0 µM treatment in H1975 (Fig. 5E). These data confirmed that selenite induced ferroptosis and UPR in both H1975 and H358 but more markedly in the EGFR-mutant H1975.

### Interaction between sodium selenite and targeted therapy

**Fig. 6:**
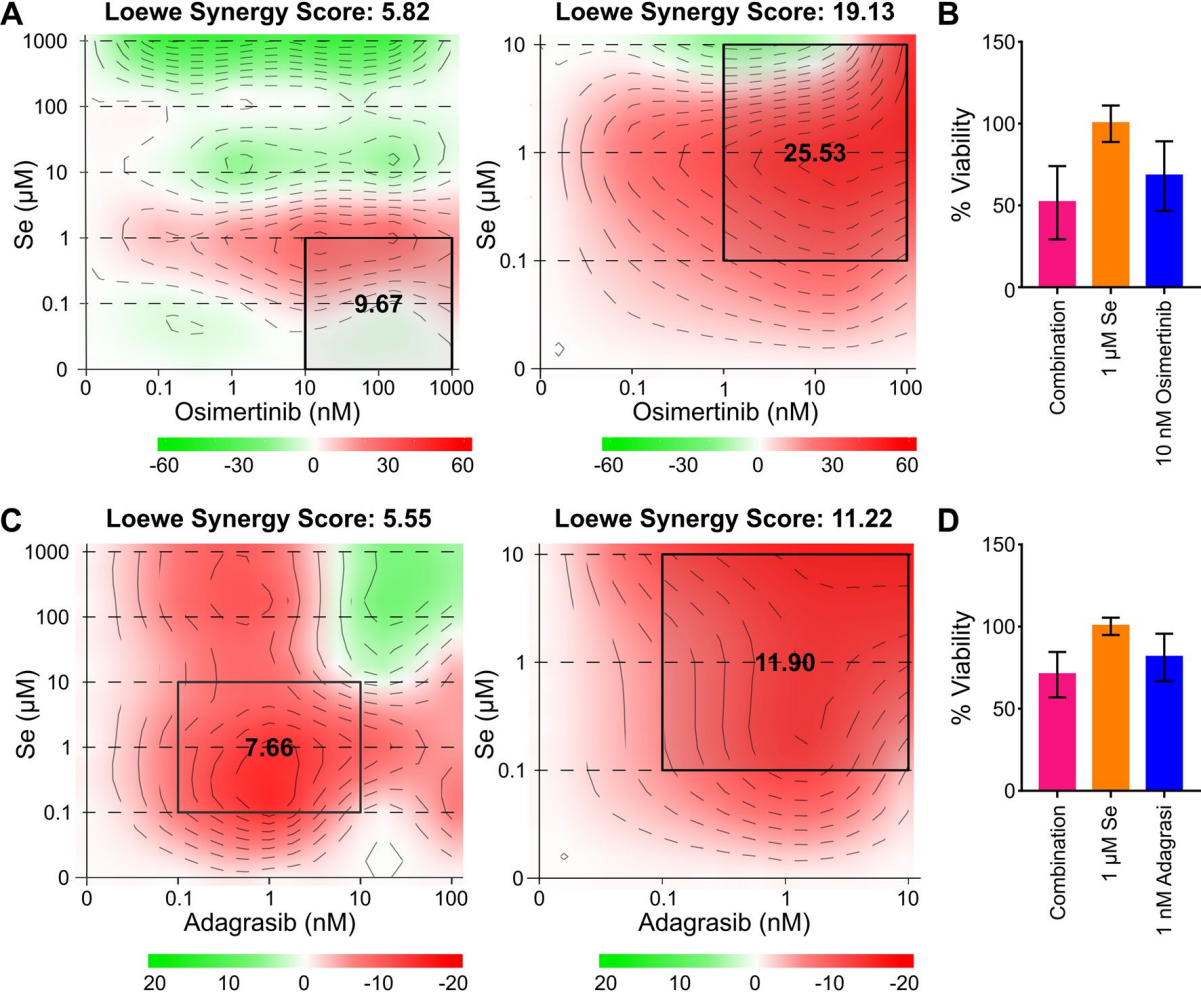
Low-dose selenite treatment synergizes with targeted therapy. **A** Loewe synergy scores and maps of selenite and osimertinib in H1975. The rectangles represented the most synergistic area. **B** % viability of single-agent 1 µM Se, 10 nM osimertinib, and their combination. **C** Loewe synergy scores and maps of selenite and adagrasib in H358. The rectangles represented the most synergistic areas. **D** % viability of single-agent 1 µM Se, 1 nM adagrasib and their combination

Our findings confirmed that selenite serves as dual apoptosis and ferroptotic agent, best seen in H1975; we thus explore its potential as a drug candidate to synergize with drugs of the current standard of care, EGFR TKI and KRAS inhibitors. Loewe synergy scores for interaction between selenite with osimertinib in H1975 cells were 5.82 at a high dose and 19.13 when limited at a low dose (Fig. 6A). Cell viability when treated with 1 µM selenite and 10 nM osimertinib was lower than that of the single agents (Fig. 6B). The most synergistic area with a synergy score of 25.53 corresponded to 1–100 nM osimertinib and 0.1–10 µM selenite. The same score between selenite and adagrasib in H358 was 5.55 at a high dose and 11.2 at a low dose (Fig. 6C). Cell viability when treated with 1 µM selenite and 1 nM adagrasib was lower than that of the single agents (Fig. 6D).The most synergistic area had a score of 11.9 and corresponded to 0.1–10 nM adagrasib and 0.1–10 µM selenite. In summary, low-dose selenite at 0.1–10 µM concentration synergizes with osimertinib and adagrasib, but the synergism is again more marked in EGFR-mutant H1975.

## Discussion

Selenite, an essential trace element, has been proposed to be an antineoplastic agent through multiple mechanisms. Only recently, selenite in combination with chemotherapy demonstrated clinical response at a tolerable dose in phase 1 SECAR trial in patients refractory to up to four established treatments [36]. However, its role in targeted therapy for LUAD carrying different oncogenic signatures remained uncertain. Meanwhile, specifically for LUAD, the underlying mechanisms of selenite to apoptosis, demethylation, and ferroptosis are largely unknown. To the best of our understanding, this study is the first to identify the role of selenium in LUAD cells harboring oncogenic driver mutations.

We confirmed that selenite acted as a dual apoptosis and ferroptotic agent in EGFR^L858R/T790M^ H1975 and KRAS^G12C^ H358 cells, through RNA-sequencing, RT-qPCR, western blot, and flow cytometry. For apoptosis, selenite treatment resulted in phosphorylation of p38 MAPK and upregulation of the ATF4-DDIT3 axis in UPR (Figs. 3C, 5C). Our result of selenite-induced ER stress remained consistent with other studies on normal tissues and malignant tissues [7–13]. The UPR is caused by abnormal protein folding in ER and can result in apoptotic cell death [39]. *ATF4* initiates the transcription of *DDIT3*, another pro-apoptotic transcription factor, to induce the expression of BH3-only proteins, including BIM and NOXA [19, 22, 23]. Meanwhile, the phosphorylation of p38 MAPK was shown to be essential for the switch of cell fate with activated UPR, from autophagy to apoptosis, by enhancing the docking of ATF4 to the promoter of DDIT3 [40]. These findings explained how selenite induced apoptosis as demonstrated by increased cleavage of PARP, caspase 3, and upregulation of cytochrome C and BH3-only proteins (Fig. 1C, D).

For ferroptosis, both H1975 and H358 displayed dysregulated transcriptome seen in ferroptosis, despite the lower dose of selenite in H358. However, the magnitude of increase in lipid peroxidation was more marked in H1975 than that of H358 at both 2.5 µM and 5.0 µM selenite (Fig. 5D, E). In addition, enrichment analysis under the GO terms showed that enriched terms in H1975 were highly related to glutathione synthesis and ER stress, while that seen in H358 remained inconsistent. We hypothesized that selenite acted more like a traditional chemotherapeutic agent than a ferroptosis inducer in H358 as seen with perturbed DNA repair biological processes or microtubules-related cellular components (Fig. 4A, B). Indeed, KRAS-driven lung cancer has a greater resistance to ferroptosis owing to a reprogrammed lipid metabolism by a higher level of acyl-coenzyme A synthetase long-chain family member 3 (ACSL3) expression [16]. In this study, the level of ACSL3 mRNA expression in H358 was significantly higher than that in H1975 before and after selenite treatment, although its expression also decreased slightly after the introduction of selenite (Additional file 1: Fig. S1D) [41]. In contrast, EGFR mutations in non-small cell lung cancer cells render them more susceptible to SLC7A11 inhibition or cysteine deprivation because intracellular cysteine promoted survival EGFR-mutant cells independent of GSH-relater redox balance [42]. The inhibition of SLC7A11 removes this favorable metabolic signature while introducing additional oxidative stress. Therefore, ER stress and ferroptosis-related signature were significantly enriched in H1975, but other antineoplastic effects of selenite, such as causing DNA damages, dominated the transcriptomic landscape in H358.

Our data also showed that low-dose selenite of 0.1–10 nM range synergized with osimertinib, a third-generation EGFR TKI, and adagrasib, a KRAS G12C inhibitor. Because the synergism was more marked in EGFR-mutant H1975 than the KRAS-mutant H358, we found it consistent with the ferroptotic effect of selenite in the two cell lines. Theoretically, EGFR inhibition disfavors ferroptosis because MAPK signaling is required for ferroptosis, while its inhibition by EGFR or ERK inhibitors aborts the process of ferroptosis in lung cancer cells [42, 43]. However, ROS, such as that in ferroptosis, disfavors the emergence of resistance to osimertinib, while the addition of ROS scavenger, N-acetylcysteine, to osimertinib increases the proportion of cycling persister cells [44]. In our multiple-dose drug combination studies, maximal single-agent inhibition of only around 60% demonstrated a subgroup of osimertinib-resistant cells in our culture, likely owing to heterogeneity (Additional file 1: Fig. S1E). We hypothesize that selenite acts on these resistant cells where MAPK signaling was insufficiently or was not inhibited by osimertinib to enhance its therapeutic effect. Therefore, it would be interesting to know if the oxidative stress exerted by selenite can reduce the emergence of osimertinib resistance in a long run.

In terms of its epigenetic property, only being synergistic at low doses, selenite resembles the dose-dependent therapeutic profile of other DNA-demethylating agents, such as azacytidine and decitabine [45, 46]. In our study, while the expression of DNA methylation machinery was altered, we did not observe significant enrichment in related processes after RNA sequencing, reflecting that the DNA demethylation property of selenite was not its predominant mechanism of action in LUAD cells harboring oncogenic mutations (Fig. 4). However, our treatment duration was limited to 72 h only in contrast to the significantly longer duration of 7–14 days in most studies demonstrating the demethylating effect of selenite [26–30]. This finding suggests that selenite is a less potent DNA demethylator than traditional demethylating agents because it can only achieve demethylation at a much longer duration of treatment. Meanwhile, UPR alone can lead to diverse cellular outcome, including recovery from cellular stress or as in the case of selenite, cell death [47]. It is unlikely that UPR alone is sufficient to trigger cellular death and epigenetic interference should contribute to the antineoplastic effect of selenite.

Lastly, it would be intriguing to know whether selenite provides a greater benefit as an adjunctive or neoadjuvant treatment to targeted therapy. The ex vivo experiment in the SECAR trial demonstrated that selenite sensitization with subsequent chemotherapy of carboplatin alone, gemcitabine alone, or a combination of carboplatin and gemcitabine induced greater cytotoxicity than that of chemotherapy alone [36].

In summary, selenite is a potent dual apoptotic and ferroptotic agent against LUAD along with the activation of UPR. Our data demonstrated that EGFR-mutant H1975 was more susceptible to selenite-induced H358. Selenite also synergized osimertinib better in H1975 than adagrasib in H358. These data provided preclinical evidence that selenite is a potential drug candidate for the treatment of especially EGFR mutation-positive lung cancer. Further work is warranted to explore the long-term effect of selenite and its combination with TKI on EGFR mutation-positive lung cancer.

## Materials and methods

### Reagents

Sodium selenite (Sigma Chemical Co., St Louis, MO) was dissolved in phosphate-buffered solution (PBS) at 200 mM, aliquoted, and stored at − 30 °C. Adagrasib (MedChemExpress, Monmouth Junction, NJ) was dissolved in DMSO at 20 mM, aliquoted, and stored at − 80 °C. Osimertinib (Selleck Chemicals, Houston, TX) was dissolved in DMSO at 50 mM, aliquoted, and stored at − 80 °C.

### Cell culture

LUAD cell lines (H358, H1975) were obtained from ATCC, cultured in RPMI 1640 media, containing 10% heat-inactivated fetal bovine serum, 100 g/mL streptomycin and 100 units/mL penicillin (Gibco, Waltham, MA). Cells were maintained at 37 °C in humidified, 5% CO_2_ atmosphere.

### Cell viability assay

The effects of selenite on the proliferation of LUAD cell lines were evaluated by an MTS assay (Promega Co., Madison, WI). 5000 cells per well were seeded in 96-well microtiter plates. Cells were treated after 24 h with fresh growth media with different concentrations of selenite (0–1000 μM) in triplicates. Cell proliferation at 24, 48, and 72 h was then examined as manufacturer protocol.

### Cell cycle analysis

Cells were treated in three biological repeats for 72 h, collected using 0.25% trypsin in EDTA and washed twice in PBS. Cells were fixed in ice-cold 70% ethanol, washed in ice-cold PBS, and stained in 500 µL PI/Triton X-100 solution, containing 100 µg/mL DNAase-free RNAse A (Sigma Chemical Co.), 50 µg/mL propidium iodide (Sigma Chemical Co.), and 0.25% (v/v) Triton X-100 (Sigma Chemical Co.) in PBS, for 1.5 h at room temperature in dark. Cells are then washed in cold PBS and analyzed by a flow cytometer (BD Accuri C6, BD Biosciences, Franklin Lakes, NJ). Data from at least 10,000 events per sample were collected and presented as proportions of the cells in G0/G1, S, and G2/M phases using ModFit LT 5.0 software (Verity Software House, Topsham, ME). Experiments were repeated in three triplicates.

### Western blot

Cell pellets were collected using 0.25% trypsin in EDTA and stored at − 80 °C until used. They were lysed in RIPA buffer supplemented with protease and phosphatase inhibitors and stored at − 80 °C until analysis by western blot. Protein concentration was measured by Pierce™ BCA Protein Assay Kit (Thermo Fisher Scientific, Waltham, MA) using the manufacturer’s protocol. The lysate was electrophoresed in SDS-PAGE and then transferred onto nitrocellulose membranes (GE Healthcare, Chicago, IL). After transfer, the membrane was incubated in freshly prepared 5% non-fat dry milk (BioRad, Hercules, CA) for 1 h and then incubated against the primary antibodies listed in Additional file 2: Table S1a, diluted in 5% non-fat dry milk, overnight at 4 °C. The membranes were then incubated in secondary antibodies, listed in Additional file 2: Table S1b, and then developed using the ECL method (GE Healthcare) and visualized using ChemiDoc MP image visualizer (BioRad). Quantification was performed using Image J.

### Reverse transcription-polymerase chain reaction (qRT-PCR)

Total RNA was extracted using AllPrep DNA/RNA Mini Kit (Qiagen, Hilden, Germany). Reverse transcription was performed using the GeneAmp system. Subsequent qPCR was carried out according to the manufacturer’s protocol (StepOne™ system; Applied Biosystems, Waltham, MA) using SYBR Green master mix (Applied Biosystems) and GAPDH as an internal control. The primers used are listed in Additional file 2: Table S2. Means and standard deviations were obtained from three triplicates.

### RNA-sequencing

H1975 and H358 were treated in 5.0 µM and 2.5 µM in triplicate for 72 h, respectively, to achieve DNMT1 downregulation. Total RNA was extracted using AllPrep DNA/RNA Mini Kit (Qiagen). RNA was analyzed by Bioanalyzer (Agilent 2100). RNA integrity number of analyzed samples and ribosomal RNA 28S-18S ratio spanned from 8.4 to 9.7 and 1.8 to 2.0, respectively. mRNA library was prepared following standard BGI protocol. Samples are then sequenced on the DNBseq™ platform at BGI (Shenzhen, China) to produce around 40–50 total clean reads of 150 bp and around 6.0 -7.5 GB of total clean bases, whose clean reads Q20 and Q30 are above 95% and 85%, respectively. SOAPnuke (v1.5.2) was adopted for filtering. RefSeq Assembly GRCh38.p12 was the reference transcriptome where alignment was performed using Hierarchical Indexing for Spliced Alignment of Transcripts (v2.0.4) and mapping of the clean reads was performed using Bowtie2 (v2.2.5) for mapping the clean reads. Differentially expressed gene was detected using DEseq2 method with q-value <  = 0.05. Genes at the transcript resolution with |log_2_FC value|> = 0.5 were plotted in a cluster heatmap using pheatmap standardized by z-score using default parameters. The phyper function in R software was used to perform the enrichment analysis according to the kegg_pathway annotation, and DEGs with |log_2_FC value|> = 1.5 were selected for go_f, go_p, and go_c enrichment. Representative KEGG pathways with q-value < 0.10 were presented in bubble charts. Gene set enrichment analysis was performed using GSEA software at the gene resolution because transcript-level annotations are not available [48, 49]. Validated ferroptosis inducers, suppressors, and markers on FerrDb were matched to sequencing results at the gene resolution [38].

### Measurement of lipid peroxidation

Cells were probed with C11-BODIPY at a final concentration of 1.5 μmol/L for 30 min and then analyzed using a flow cytometer.

### Multiple-dose drug combination studies

The drug interactions were evaluated by an MTS assay (Promega Co.). 5000 cells per well were seeded in 96-well microtiter plates. After 24 h, cells were treated with varying drug dosages in triplicates. Cell proliferation at 72 h was examined as manufacturer protocol. Data from at least three replicates were then analyzed in SynergyFinder 2.0 [50].

### Statistical Analysis

Two-way ANOVA was performed using Graphpad Prism with p < 0.05 considered as statistical significance (*P ≤ 0.05; **P ≤ 0.01; ***P ≤ 0.001; ****P ≤ 0.0001). For RT-qPCR, two-way ANOVA was performed based on ddCT values.

## Supplementary Information

**Additional file 1**: **Fig. S1** **A** Western blot analysis showed that selenite did not alter the cleavage of caspase 8. **B **Volcano plots of the transcriptome of H1975 treated with or without 5µM selenite and H358 treated with or without 2.5µM Se at the transcript resolution. **C **GSEA analysis of the upregulated DEGs at a gene resolution of both cell lines revealed alteration in cell cycle checkpoints and ATR signaling. **D** ASCL3 expression in transcript per million from RNA-seq data was higher in H358 than H1975 before and after selenite treatment. **E** Dose-response curve of single-agent osimertinib in H1975 showed maximal inhibition of around 60%.

**Additional file 2**: **Table S1** Antibodies used in this study. **Table S2** Primers for RT-qPCR.

## Data Availability

The datasets used and/or analyzed during the current study are available from the corresponding author on reasonable request.
