# Supplementary figures and images for: Selenite as a dual apoptotic and ferroptotic agent synergizes with EGFR and KRAS inhibitors with epigenetic interference
Source: Clin Epigenetics. 2023 Mar 2;15:36. doi: 10.1186/s13148-023-01454-4 (PMC9983273; doi:10.1186/s13148-023-01454-4)

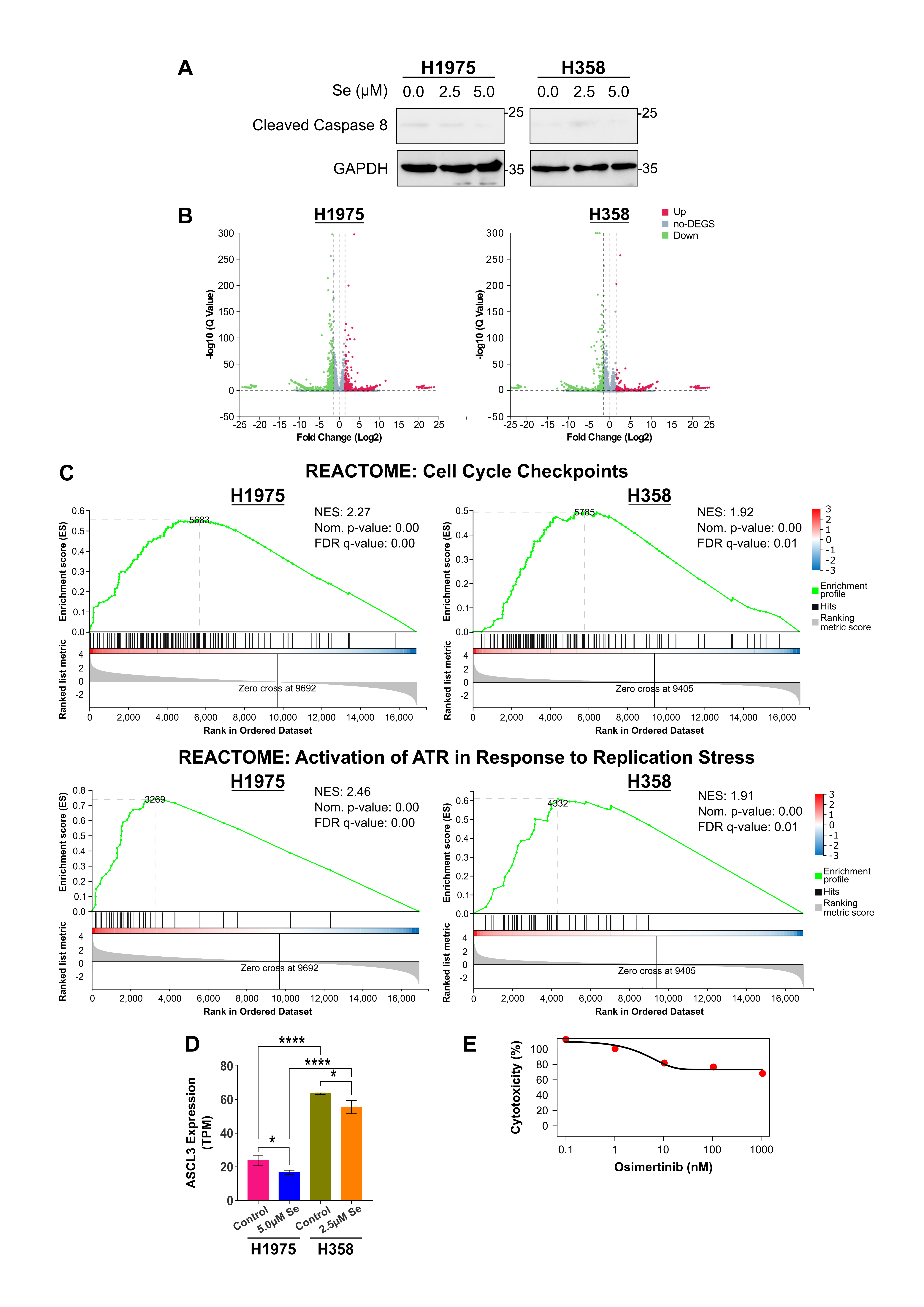

Supplement: Supplementary file 1 — Additional file 1: Fig. S1 A Western blot analysis showed that selenite did not alter the cleavage of caspase 8. B Volcano plots of the transcriptome of H1975 treated with or without 5µM selenite and H358 treated with or without 2.5µM Se at the transcript resolution. C GSEA analysis of the upregulated DEGs at a gene resolution of both cell lines revealed alteration in cell cycle checkpoints and ATR signaling. D ASCL3 expression in transcript per million from RNA-seq data was higher in H358 than H1975 before and after selenite treatment. E Dose-response curve of single-agent osimertinib in H1975 showed maximal inhibition of around 60%. [file 13148_2023_1454_MOESM1_ESM.jpg]
